# Supplementary material for: Long COVID Classification: Findings from a Clustering Analysis in the Predi-COVID Cohort Study
Source: Int J Environ Res Public Health. 2022 Nov 30;19(23):16018. doi: 10.3390/ijerph192316018 (PMC9740149; doi:10.3390/ijerph192316018)
Supplement: Supplementary file 1 [file ijerph-19-16018-s001.zip › ijerph-2034257-supplementary.pdf]

Supplementary Materials

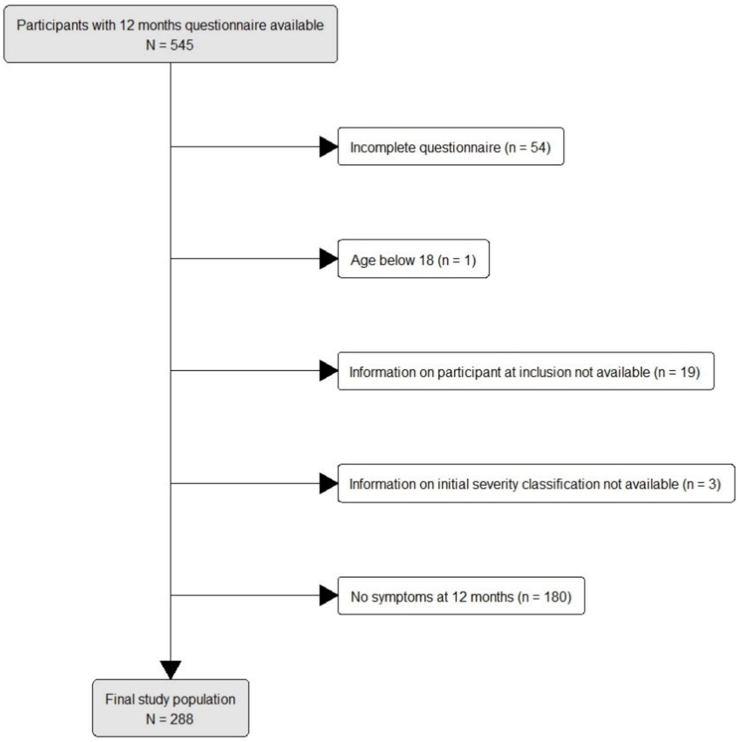

Figure S1. Flowchart of participants included in the analyses (N = 288).

Table S1. Full list of persisting symptoms considered in the 12-months questionnaire.

**Ear/Nose/Throat Symptoms**	**Neurological and Ocular Symptoms**	**General Symptoms**	**Cardio-Respiratory Symptoms or Diseases**	**Gastrointestinal Symptoms**	**Vascular and Ganglionic Symptoms or Diseases**	**Urinary Symptoms**	**Skin Symptoms**
Loss of taste	Tremors	Fatigue	Shortness breath	Nausea	Hypertension	Urinary pain	Skin rashes
Loss of smell	Headaches	Irritability	Chest tightness	Vomiting	Hypotension	Urinary infections	Dry skin
Runny nose, cold or rhinitis	Migraines	Anxiety	Dry cough	Diarrhea	Adenopathies	Dialysis	Blue fingers
Sinus pain	Mental confusion	Depression	Fatty cough	Heartburn	Circulation disorders		
Ear pain	Malaise	Sweating	Tachycardia	Abdominal pain	Hematoma		
Sore throat	Convulsions	Fever	Arrhythmia				
	Balance	Loss of appetite	Myocarditis				
	Memory	Loss weight	Heart failure				
	Fatigue in eyes	Thirst	Burning chest				
	Hallucinations	Ants	Chest pain				
	Sensitivity to light	Muscle pain (upper limbs)	Wheezing				
	Conjunctivitis	Muscle pain (lower limbs)	Coughing blood				
		Back pain					
		Allergy					
		Loss hair					
		Difficulty walking					

Table S2. Full list of features included in the clustering.

Sociodemographic Characteristics and Initial Severity Classification	Inclusion at home or at hospital
	Gender
	Age
	BMI
	Weight loss in last 6 months
	Smoking status
	Classification severity initial illness
	Blood type
Comorbidities	Hypertension
	Cardiac diseases
	Pulmonary diseases
	Asthma
	Renal diseases
	Hepatic diseases
	Neurological diseases
	Cancer
	Hematological diseases
	Obesity
	Diabetes
	Rheumatological diseases
	Malnutrition
	COPD
Symptoms at inclusion	Fever
	Cough
	Cough_sputum
	Cough hemoptysis
	Sore throat
	Rhinorrhea
	Earache
	Wheezing
	Chest_pain
	Myalgia
	Arthralgia
	Fatigue
	Dyspnea
	Chest tightness
	Cephalea
	Confusion
	Abdominal pain
	Nausea
	Diarrhea
	Conjunctivitis
	Skin rash
	Lymphadenopathy
	Fall
	Hemorrhage

Persisting symptoms at 12 months by categories	Ear Nose Throat (ENT) symptoms<br>Neurological symptoms<br>General symptoms<br>Cardio-respiratory symptoms<br>Gastrointestinal symptoms<br>Vascular symptoms<br>Urinary symptoms<br>Skin symptoms
Quality of life	Could not envisage coping with symptoms long term N(%)<br>PSQI score<br>VQ11 global score<br>VQ11 Functional component score<br>VQ11 Psychological component score<br>VQ11 Relational component score
